# Supplementary material for: Two-dimensional lead halide perovskite lateral homojunctions enabled by phase pinning
Source: Nat Commun. 2024 Apr 11;15:3164. doi: 10.1038/s41467-024-47406-1 (PMC11009245; doi:10.1038/s41467-024-47406-1)
Supplement: Supplementary file 1 — Supplementary Information [file 41467_2024_47406_MOESM1_ESM.pdf]

Supplementary information for

Two-Dimensional Lead Halide Perovskite Lateral Homojunctions Enabled by Phase Pinning

*Huilong Hong[1 #], Songhao Guo[2 #], Leyang Jin[1], Yuhong Mao[2], Yuguang Chen[1], Jiazhen Gu[1], Shaochuang Chen[1], Xu Huang[1], Yan Guan[1], Xiaotong Li[3], Yan Li[1], Xujie Lü[2] *, and Yongping Fu[1] **

[1] Beijing National Laboratory for Molecular Science, College of Chemistry and Molecular Engineering, Peking University, Beijing 100871, China

[2] Center for High Pressure Science and Technology Advanced Research, Shanghai 201203, China

[3] Department of Chemistry & Organic and Carbon Electronics Laboratories, North Carolina State University, Raleigh, NC, 27695, USA

[#] These authors contribute equally

* Corresponding authors: Email: xujie.lu@hpstar.ac.cn; yfu@pku.edu.cn

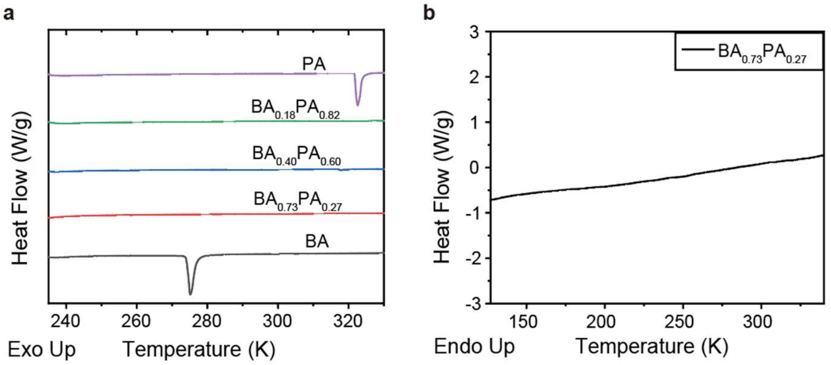

Supplementary Fig. 1. Differential scanning calorimetry. (a) Differential scanning calorimetry of $(BA)_2PbI_4$, $(PA)_2PbI_4$, and three doped $(BA_{1-x}PA_x)_2PbI_4$ in the range of 235-330 K. $(BA)_2PbI_4$ and $(PA)_2PbI_4$ exhibited peaks at 275 K and 324 K, respectively, corresponding to the order-disorder phase transition. In contrast, the three doped $(BA_{1-x}PA_x)_2PbI_4$ showed flat baselines between 235-330 K, signifying no phase transition. (b) DSC of $(BA_{0.73}PA_{0.27})_2PbI_4$ in the range of 130-330 K.

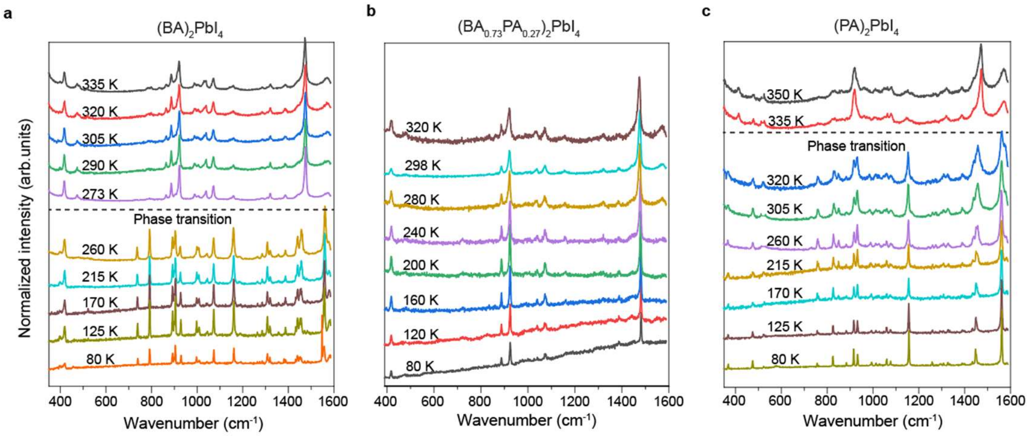

Supplementary Fig. 2. Temperature-dependent Raman spectra. Temperature-dependent Raman spectra for vibration modes of the organic cations for (a) $(BA)_2PbI_4$, (b) $(BA_{0.73}PA_{0.27})_2PbI_4$, and (c) $(PA)_2PbI_4$.

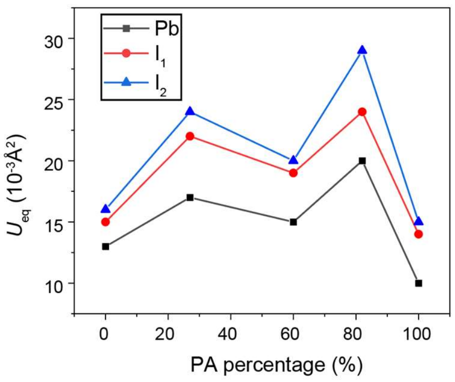

Supplementary Fig. 3. Thermal vibration analysis. Equivalent isotropic displacement parameters (U_{eq}) of lead and iodide in $(BA_{1-x}PA_x)_2PbI_4$ with different dopant ratios at 80 K. Iodine in and out of the plane are denoted by I_1 and I_2, respectively.

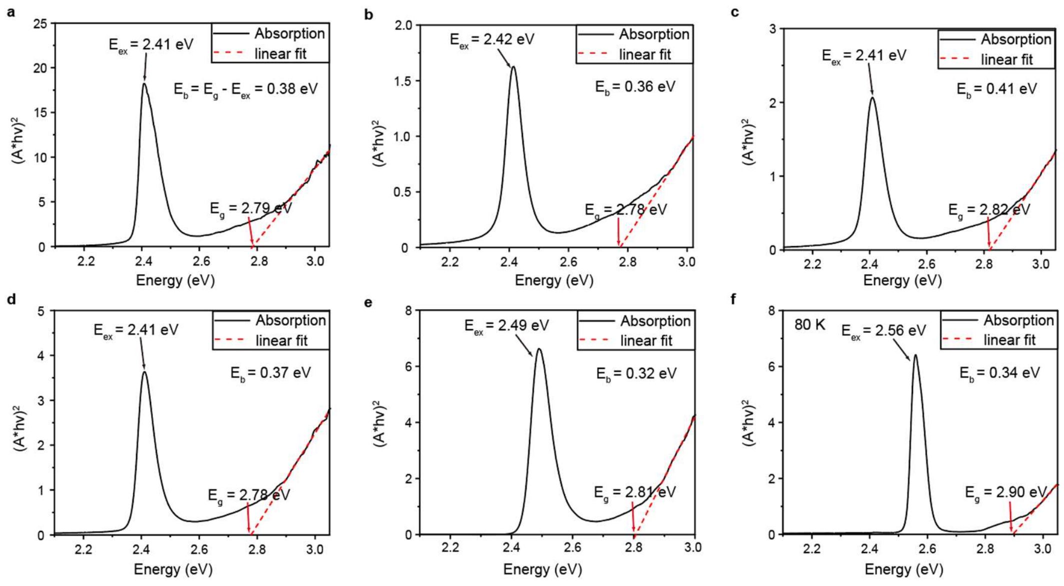

Supplementary Fig. 4. Absorption spectra of various structures. Absorption spectra of (a) $(BA)_2PbI_4$, (b) $(BA_{0.73}PA_{0.27})_2PbI_4$, (c) $(BA_{0.40}PA_{0.60})_2PbI_4$, (d) $(BA_{0.18}PA_{0.82})_2PbI_4$, and (e) $(PA)_2PbI_4$ under ambient condition. (f) Absorption spectra of the ordered phase $(BA)_2PbI_4$ at 80 K. At room temperature, the doped structures $(BA_{1-x}PA_x)_2PbI_4$ and disordered $(BA)_2PbI_4$ exhibit similar exciton binding energies, approximately 0.38 eV, aligning with their isostructural phases. In its ordered phase at room temperature, $(PA)_2PbI_4$ exhibits an exciton binding energy of around 0.32 eV. The ordered $(BA)_2PbI_4$ at 80 K exhibits an exciton binding energy of about 0.34 eV.

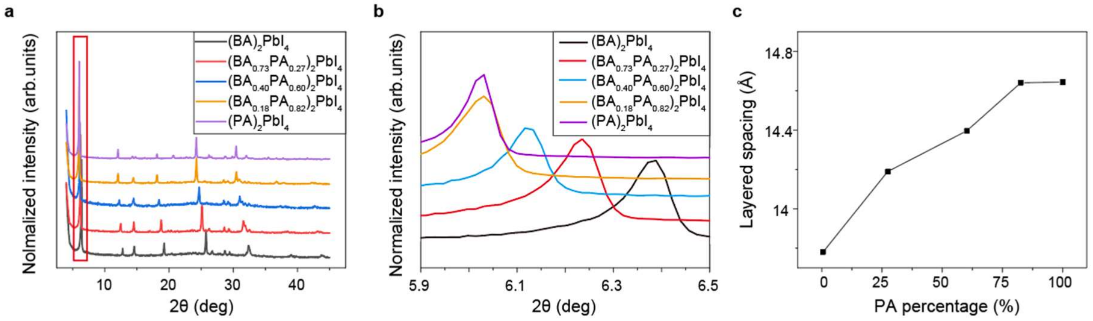

Supplementary Fig. 5. Powder X-ray diffraction analysis. (a) Powder X-ray diffraction patterns of $(BA)_2PbI_4$, $(BA_{1-x}PA_x)_2PbI_4$, and $(PA)_2PbI_4$, showing the gradual shift of the diffraction peaks. (b) Zoom on the diffraction peaks at 2θ ~ 6º in panel (a). (c) Interlayer spacing distance of $(BA_{1-x}PA_x)_2PbI_4$ determined from single-crystal structures plotted versus the x value.

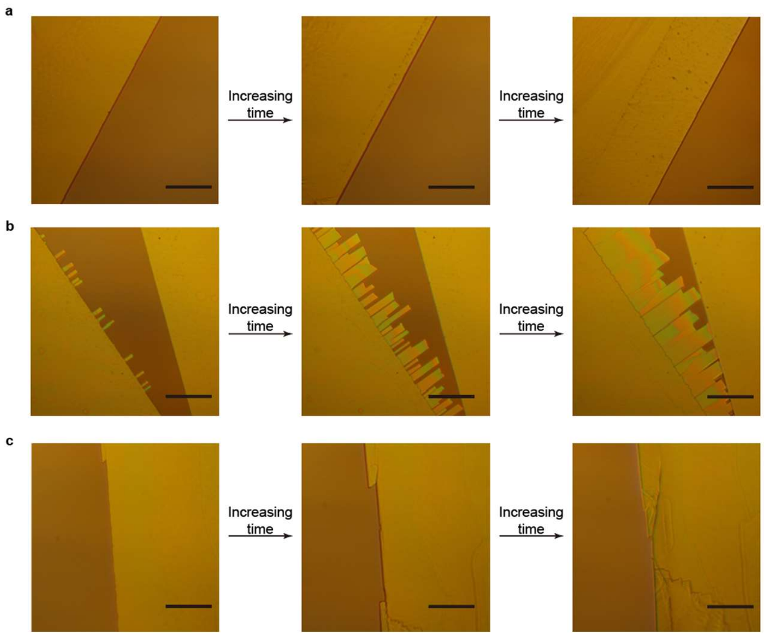

Supplementary Fig. 6. Optical images of $(BA)_2PbI_4$−$(BA_{0.8}PA_{0.2})_2PbI_4$ homojunctions showing three types of growth process. (a) Uniform epitaxial growth of $(BA_{0.8}PA_{0.2})_2PbI_4$ on the edge of $(BA)_2PbI_4$. (b) Multiple nucleation sites on the edge of $(BA)_2PbI_4$ lead to epitaxial growth of separated $(BA_{0.8}PA_{0.2})_2PbI_4$ flakes. As the flakes grew, they gradually merged together. (c) Epitaxial growth of $(BA_{0.8}PA_{0.2})_2PbI_4$ along the edge of $(BA)_2PbI_4$. Scale bar = 50 μm.

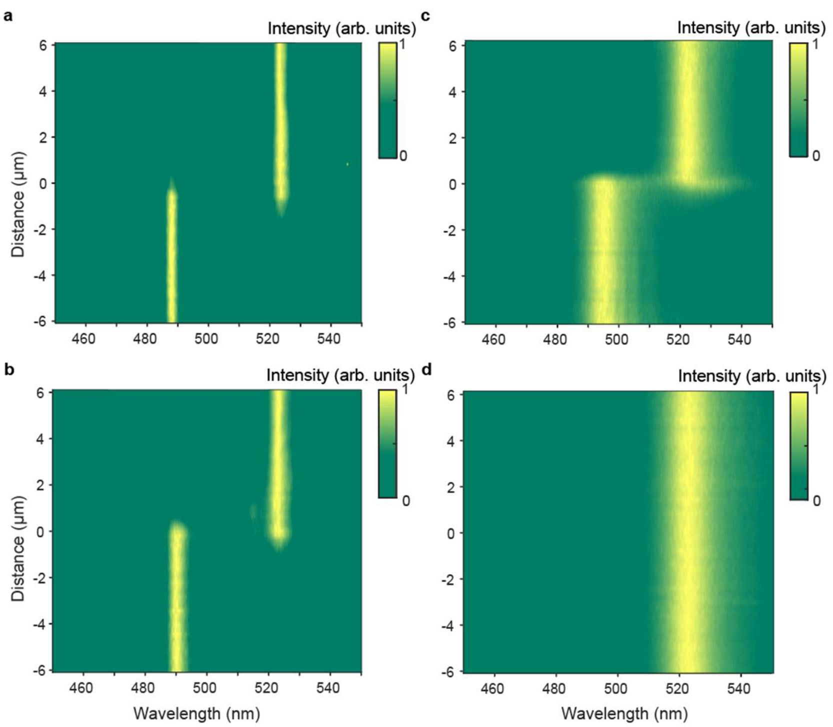

Supplementary Fig. 7. Spatially resolved PL spectra under low temperatures. Spatially resolved PL spectra of a $(BA)_2PbI_4$−$(BA_{0.8}PA_{0.2})_2PbI_4$ along the direction across the interface at 80 (a), 140 (b), 230 (c), and 298 K (d).

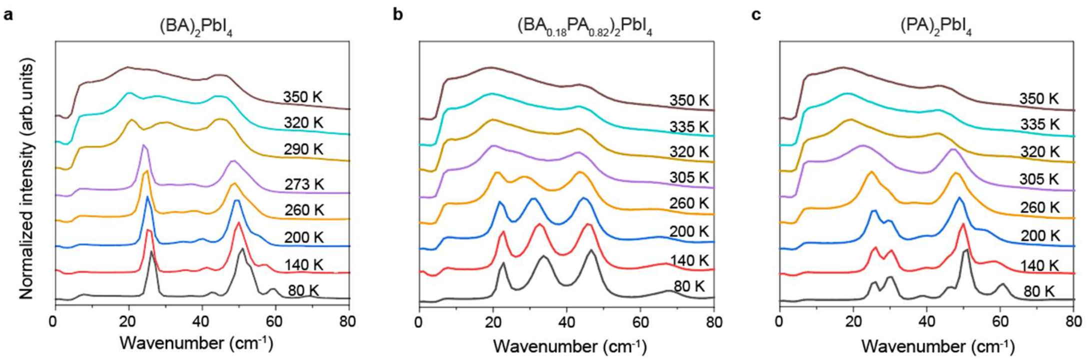

Supplementary Fig. 8. Low-frequency temperature-dependent Raman spectra. Low-frequency temperature-dependent Raman spectra of (a) $(BA)_2PbI_4$, (b) $(BA_{0.18}PA_{0.82})_2PbI_4$ and (c) $(PA)_2PbI_4$.

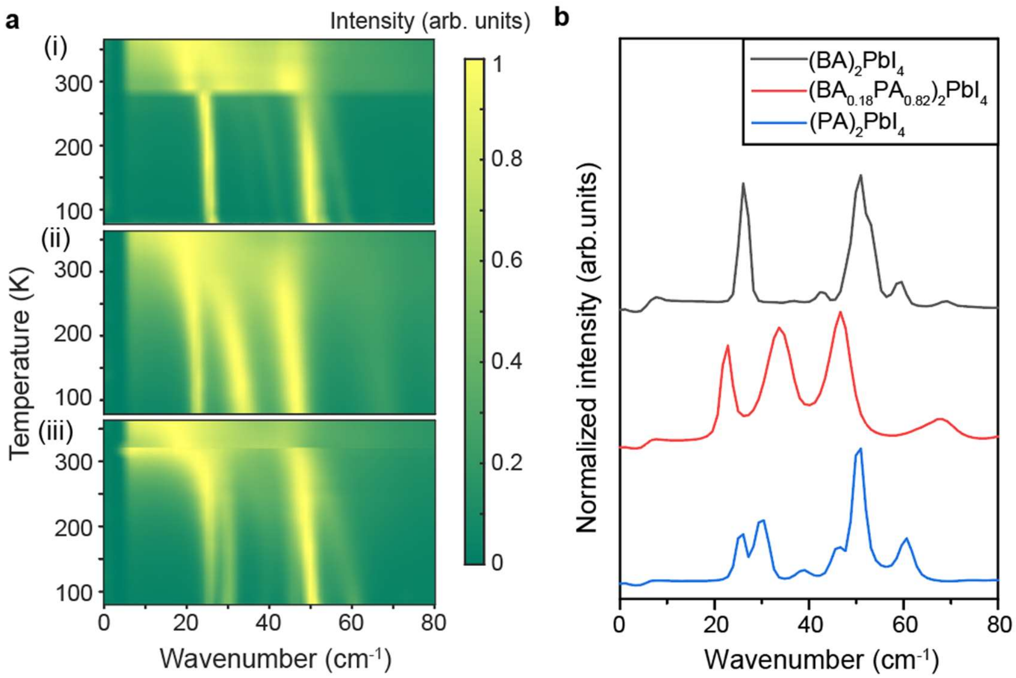

Supplementary Fig. 9. The comparison of low-frequency Raman spectra. (a) Temperature-dependent Raman spectra of $(BA)_2PbI_4$ (i), $(BA_{0.18}PA_{0.82})_2PbI_4$ (ii) and $(PA)_2PbI_4$ (iii). (b) Raman spectra of $(BA)_2PbI_4$, $(BA_{0.18}PA_{0.82})_2PbI_4$, and $(PA)_2PbI_4$ at 80 K. An additional peak splitting in $(PA)_2PbI_4$ is observable in comparison to $(BA)_2PbI_4$, particularly at around 26 cm^{-1}.

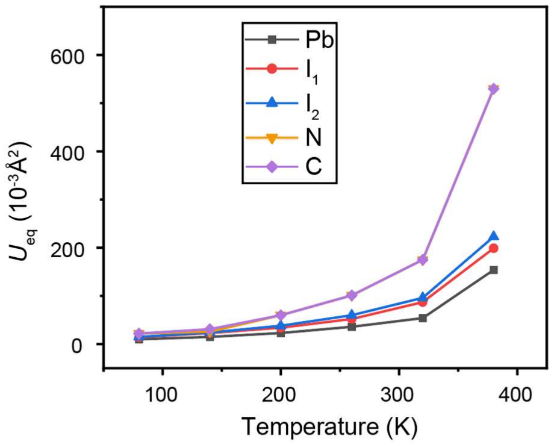

Supplementary Fig. 10. Thermal vibration analysis. Equivalent isotropic displacement parameters (U_{eq}) of $(PA)_2PbI_4$ under different temperatures.

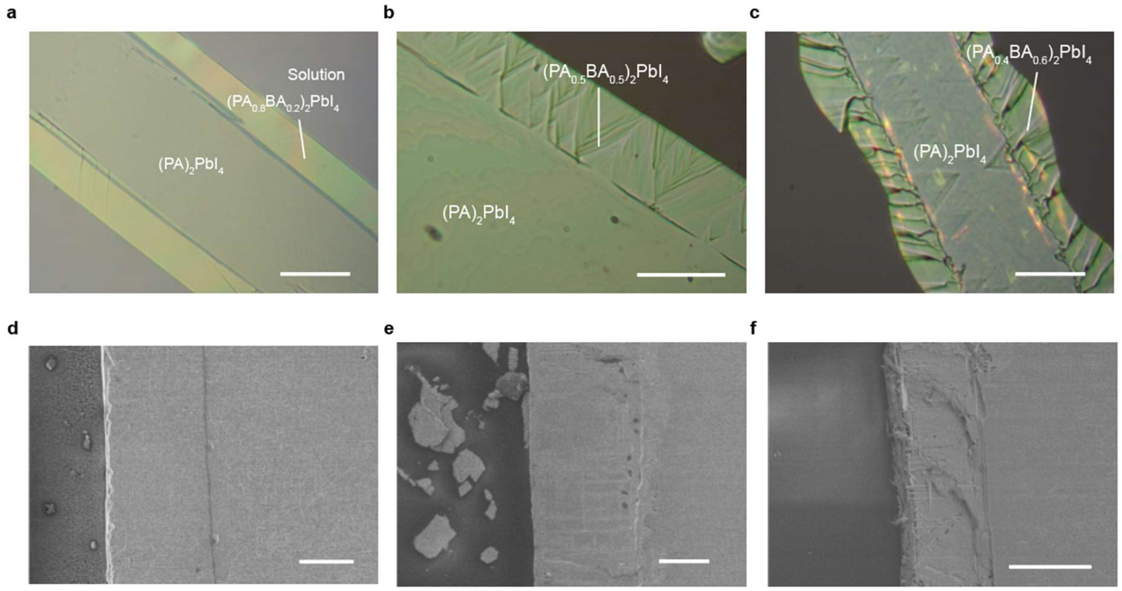

Supplementary Fig. 11. Optical and SEM images. (a) Optical image of $(PA)_2PbI_4$–$(PA_{0.8}PA_{0.2})_2PbI_4$. Scale bar = 50 μm. (b, c) Optical image of $(PA)_2PbI_4$–$(PA_{0.8}PA_{0.2})_2PbI_4$ and of $(PA)_2PbI_4$–$(PA_{0.5}PA_{0.5})_2PbI_4$, Scale bar = 20 μm. (d-f) SEM images of $(PA)_2PbI_4$–$(PA_{0.8}PA_{0.2})_2PbI_4$, $(PA)_2PbI_4$–$(PA_{0.5}PA_{0.5})_2PbI_4$ and $(PA)_2PbI_4$–$(PA_{0.4}PA_{0.6})_2PbI_4$. Scale bar = 10 μm.

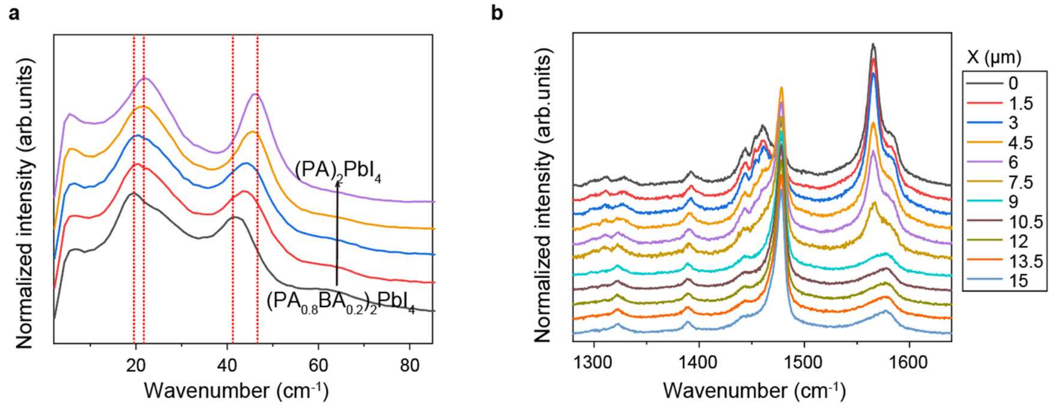

Supplementary Fig. 12. Spatially resolved Raman spectra. (a) Low-frequency and (b) high-frequency Raman spectra of $(PA)_2PbI_4$–$(PA_{0.8}BA_{0.2})_2PbI_4$ taken at multiple locations across the interface under ambient condition.

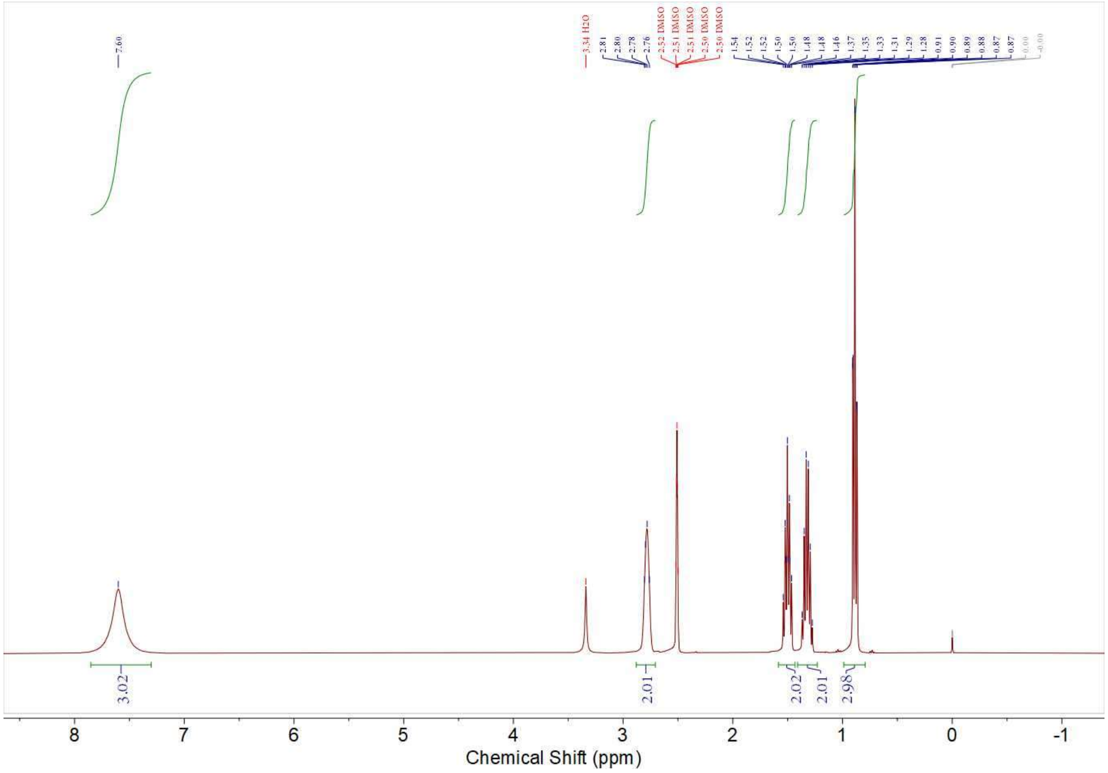

Supplementary Fig. 13 ^{1}H NMR spectrum (400 MHz, DMSO-d_6) of $(BA)_2PbI_4$.

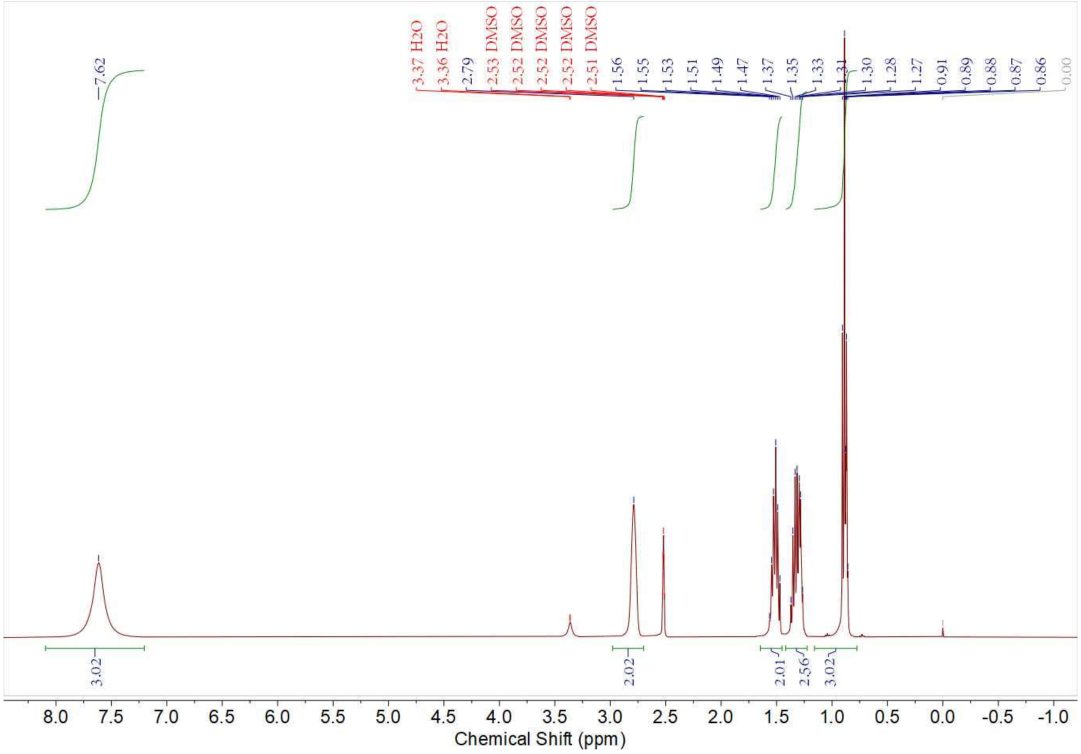

Supplementary Fig. 14 ^{1}H NMR spectrum (400 MHz, DMSO-d_6) of $(BA_{0.73}PA_{0.27})_2PbI_4$.

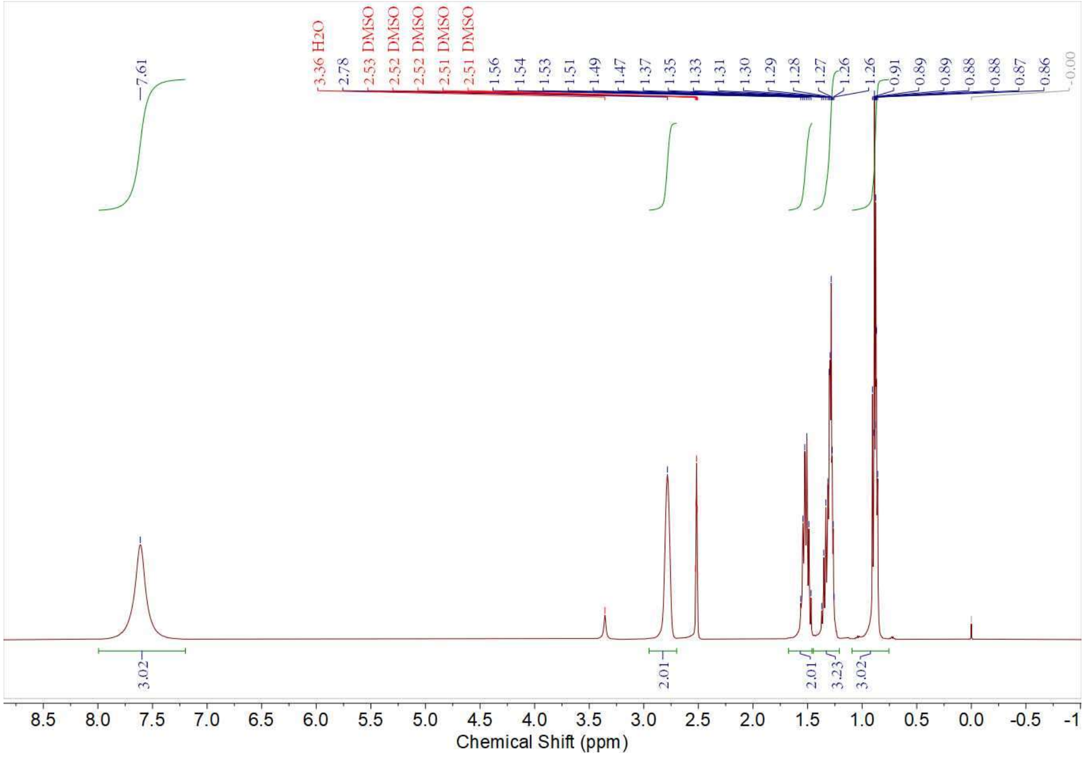

Supplementary Fig. 15 ^{1}H NMR spectrum (400 MHz, DMSO-d_6) of $(BA_{0.40}PA_{0.60})_2PbI_4$.

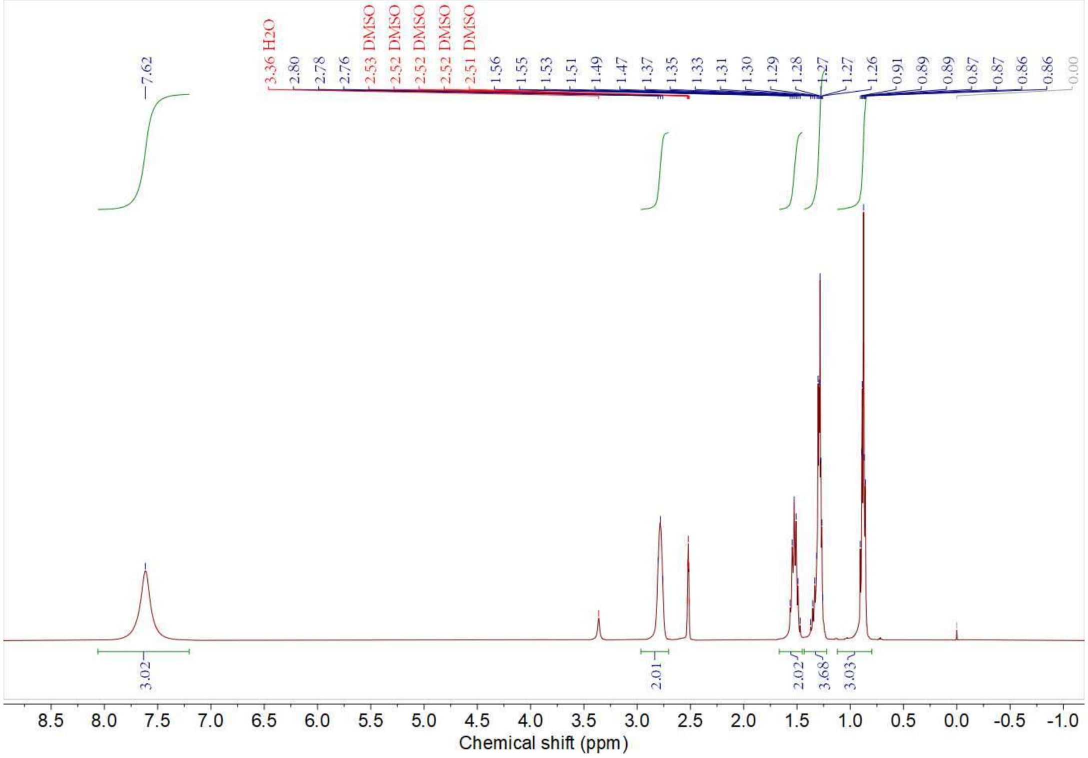

Supplementary Fig. 16 ^{1}H NMR spectrum (400 MHz, DMSO-d_6) of $(BA_{0.18}PA_{0.82})_2PbI_4$.

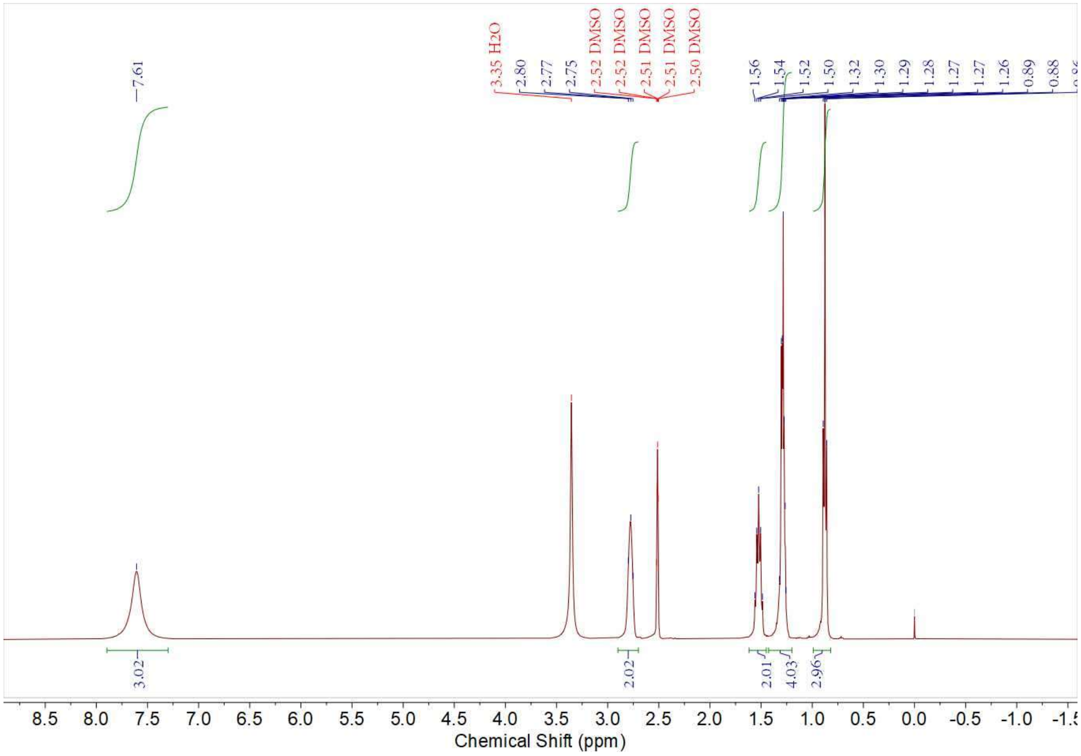

Supplementary Fig. 17 ^{1}H NMR spectrum (400 MHz, DMSO-d_6) of $(PA)_2PbI_4$.

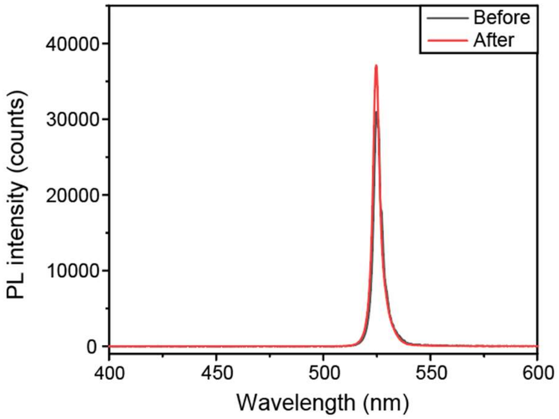

Supplementary Fig. 18. Photostability of the sample. PL spectra of $(BA)_2PbI_4$ under a 5 μW 405 nm laser exposure for 30 min at 80 K.

Supplementary Table 1. Crystal data and structural parameters of $(BA)_2PbI_4$, $(PA)_2PbI_4$, and $(PA_{1-x}BA_x)_2PbI_4$.

Chemical formula	$(BA)_2PbI_4$	$(BA_{0.73}PA_{0.27})_2PbI_4$	$(BA_{0.40}PA_{0.60})_2PbI_4$	$(BA_{0.18}PA_{0.82})_2PbI_4$	$(PA)_2PbI_4$
Temperature (K)	293	290	290	290	293
Crystal system	Orthorhombic	Orthorhombic	Orthorhombic	Orthorhombic	Orthorhombic
Space group	*Pbca*	*Pbca*	*Pbca*	*Pbca*	*P*21/*a*
a(Å)	8.8632	8.9629	8.9473	8.9761	8.6716
b(Å)	8.6816	8.8226	8.7478	8.7988	8.9297
c(Å)	27.57	28.384	28.7961	29.282	14.8805
β(deg)	90	90	90	90	100.21
Volume of unit cell($Å^3$)	2121.42	2244.50	2253.85	2312.66	1134.01
Layered spacing(Å)	13.78	14.192	14.398	14.641	14.64
Horizontal I-Pb-Pb-I dihedral angle (deg)	4.38	4.51	4.98	5.37	20.52
Layer shift factor (LSF)	(0.5,0.5)	(0.5,0.5)	(0.5,0.5)	(0.5,0.5)	(0.304,0.304)

Supplementary Table 1. (Continued)

Chemical formula	$(BA)_2PbI_4$	$(BA_{0.73}PA_{0.27})_2PbI_4$	$(BA_{0.40}PA_{0.60})_2PbI_4$	$(BA_{0.18}PA_{0.82})_2PbI_4$	$(PA)_2PbI_4$
Temperature (K)	80	80	80	80	80
Crystal system	Orthorhombic	Orthorhombic	Orthorhombic	Orthorhombic	Monoclinic
Space group	*Pbca*	*Pbca*	*Pbca*	*Pbca*	*P*21/*a*
a(Å)	8.4138	8.76	8.7827	8.8094	8.4118
b(Å)	8.984	8.6549	8.6786	8.741	9.0279
c(Å)	26.053	27.7666	28.1837	28.2138	14.7316
β(deg)	90	90	90	90	101.634
Volume of unit cell($Å^3$)	1969.34	2105.18	2148.21	2172.55	1095.75
Layered spacing(Å)	13.03	13.883	14.092	14.107	14.47
Horizontal I-Pb-Pb-I dihedral angle (deg)	22.54	2.03	2.3	2.68	22.57
Layer shift factor (LSF)	(0.5,0.5)	(0.5,0.5)	(0.5,0.5)	(0.5,0.5)	(0.351,0.351)

Supplementary Table 1. (Continued)

Chemical formula	$(PA)_2PbI_4$	$(PA)_2PbI_4$	$(PA)_2PbI_4$	$(PA)_2PbI_4$	$(PA)_2PbI_4$
Temperature (K)	380	320	260	200	140
Crystal system	Orthorhombic	Monoclinic	Monoclinic	Monoclinic	Monoclinic
Space group	*Pbca*	*P*21/*a*	*P*21/*a*	*P*21/*a*	*P*21/*a*
a(Å)	8.8352	8.7274	8.6249	8.5172	8.4524
b(Å)	8.8352	8.9802	8.9385	8.9922	9.0167
c(Å)	31.3473	14.8351	14.8877	14.8145	14.7591
β(deg)	90	99.86	100.28	100.52	101.09
Volume of unit cell($Å^3$)	2446.99	1145.50	1129.33	1115.55	1103.83
Layered spacing(Å)	15.67	14.62	14.65	14.57	14.48
Horizontal I-Pb-Pb-I dihedral angle (deg)	9.34	18.65	21.57	22.4	22.6
Layer shift factor (LSF)	(0.5,0.5)	(0.291,0.291)	(0.308,0.308)	(0.318,0.318)	(0.336,0.336)
